# Interferon-γ-inducible protein 16 (IFI16) is required for the maintenance of Epstein-Barr virus latency

**DOI:** 10.1186/s12985-017-0891-5

**Published:** 2017-11-13

**Authors:** Gina Pisano, Arunava Roy, Mairaj Ahmed Ansari, Binod Kumar, Leela Chikoti, Bala Chandran

**Affiliations:** 0000 0004 0388 7807 grid.262641.5 H.M. Bligh Cancer Research Laboratories, Department of Microbiology and Immunology, Chicago Medical School, Rosalind Franklin University of Medicine and Science, North Chicago, Illinois USA

**Keywords:** Epstein-Barr virus (EBV), Interferon-γ-inducible protein 16 (IFI16), Herpesvirus, Latency, Lytic cycle

## Abstract

**Background:**

Epstein-Barr virus (EBV) exhibits both lytic and latent (Lat. I, II, and III) phases in an infected individual. It’s during the latent phase of EBV that all EBV-associated cancers, including Burkitt’s lymphoma, nasopharyngeal carcinoma and lymphoproliferative disease arise. Interferon-γ-inducible protein 16 (IFI16) is a well-established innate immune sensor and viral transcriptional regulator involved in response to invading DNA viruses. During latency, IFI16 remains in the nucleus, in part bound to the EBV genome; however, neither its role in EBV lytic cycle or latency has been established.

**Methods:**

Short interfering RNA against IFI16 and IFI16 overexpression were used to identify the role of IFI16 in the maintenance of EBV latency I. We also studied how induction of the lytic cycle affected IFI16 using the EBV positive, latently infected Akata or MUTU-1 cell lines. Akata cells were induced with TPA and MUTU-1 cells with TGF-β up to 96 h and changes in IFI16 protein were analyzed by Western blotting and immunofluorescence microscopy. To assess the mechanism of IFI16 decrease, EBV DNA replication and late lytic transcripts were blocked using the viral DNA polymerase inhibitor phosphonoacetic acid.

**Results:**

Knockdown of IFI16 mRNA by siRNA resulted in enhanced levels of EBV lytic gene expression from all temporal gene classes, as well as an increase in the total EBV genome abundance, whereas overexpression of exogenous IFI16 reversed these effects. Furthermore, 96 h after induction of the lytic cycle with either TPA (Akata) or TGF-β (MUTU-1), IFI16 protein levels decreased up to 80% as compared to the EBV-negative cell line BJAB. Reduction in IFI16 was observed in cells expressing EBV lytic envelope glycoprotein. The decreased levels of IFI16 protein do not appear to be dependent on late lytic transcripts of EBV but suggest involvement of the immediate early, early, or a combination of both gene classes.

**Conclusions:**

Reduction of IFI16 protein levels following lytic cycle induction, as well as reactivation from latency after IFI16 mRNA knockdown suggests that IFI16 is crucial for the maintenance of EBV latency. More importantly, these results identify IFI16 as a unique host factor protein involved in the EBV lifecycle, making it a potential therapeutic target to combat EBV-related malignancies.

## Background

Epstein-Barr virus is a lymphotropic herpesvirus that establishes a lifelong infection in its host once it is acquired. EBV has been associated with several cancers, such as Burkitt’s lymphoma (BL), Hodgkin’s lymphoma, and post-transplant lymphoproliferative disease. This oncogenic potential has been attributed to EBV due to its ubiquitous presence in different tumor types and also for its ability to infect and transform human B cells in vitro resulting in an immortalized lymphoblastoid cell line [1, 2]. Like all herpesviruses, EBV has both a lytic and a latent phase; however, EBV is unique amongst other herpesviruses as it has three latencies (Lat. I, II, and III), each defined by a tightly regulated subset of genes [3]. During initial infection, EBV replicates in the oral epithelial and/or local mucosal-associated lymphoid tissue [4, 5], with over 80 proteins expressed during production of infectious virions [6]. This is referred to as the lytic or productive phase. The temporal cascade of gene expression that follows can be subdivided into immediate early (IE), early (E), late (L), and latent (Lt). Initially, the IE gene products are expressed and act as transactivator proteins required for the expression of the E genes [7]. These E genes assist in viral DNA replication, including transcription and translation of the lytic transcripts (L) encoding the viral structural proteins and Lt. genes required for persistence of the EBV genome [7]. At the culmination of EBV replication, the virus particles are packaged and released. After primary infection of epithelial cells, EBV enters B cells where latency is established and the EBV genome is maintained as extrachromosomal episomes in the host cell. Approximately one in a million B cells at any given time are latently infected with EBV, and can periodically reactivate enabling the spread and survival of the virus [8].

All EBV-associated cancers identified to date occur as a result of the virus’ latent infection. In most cases, EBV infection is asymptomatic; whereas immunosuppressed individuals may develop EBV-associated lymphoid or epithelial-derived carcinomas. During latency, EBV can evade the host immune system, regulate apoptosis and promote B cell development and survival [9, 10]. Despite evidence that host CD8^+^CTL responses keep latently infected B cells under control, even healthy individuals experience reactivation and shedding of EBV [11, 12]. To this day, host factors important for controlling the lytic to latent or latent to lytic switch of EBV are not well understood.

Interferon-γ-inducible protein 16 (IFI16) is a multifunctional and ubiquitous host protein, initially found to be constitutively expressed in human lymphoid cells [13]. Since then, IFI16 has been reported to have critical roles in antiviral immunity. Kerur, et al. first published that IFI16 acts as a nuclear innate DNA sensor upon primary infection of human dermal microvascular endothelial cells to detect the KSHV genome in the nucleus, resulting in inflammasome activation [14]. This was the first report to demonstrate that inflammasome activation can occur in the nucleus, while identifying IFI16 as the key nuclear pathogen sensor. Additional findings showed that IFI16 constitutively senses both the latent genomes of KSHV and EBV (latencies I, II, and III) to induce the inflammasome [15, 16]. Specifically, IFI16 combines with adaptor protein ASC (apoptosis-associated speck-like protein containing CARD) and effector protein procaspase-1 to activate the IFI16/ASC/procaspase-1 inflammasome following the detection of dsDNA viruses [14]. This response leads to maturation and cleavage of the proinflammatory cytokines interleukin 1β (IL-1), IL-18 and IL-33 to the infected area, ultimately helping to fight infection. Inflammasome activation occurs in the cytoplasm, requiring IFI16 acetylation and translocation from the nucleus of the infected cell to the cytoplasm [16].

IFI16 also has important roles as a viral restriction factor. For example, Lo Cigno et al. found that overexpression of IFI16 using transfection of the adenovirus-based vector AdVIFI16 severely impaired human papilloma virus 18 replication in both NIKS and U2OS cells, while reducing viral transcription [17]. Similar studies by the same group revealed that siRNA-mediated knockdown of IFI16 resulted in enhanced human cytomegalovirus (HCMV; a β-herpesvirus) replication, and overexpression in the same system yielded decreased viral load and HCMV DNA copy number [18]. Moreover, it was reported that HSV-1 has reduced viral titers/DNA replication and gene expression in the presence of IFI16, which is in part due to IFI16’s chromatin remodeling abilities [19].

Recent studies on KSHV biology expanded our understanding of IFI16’s role in antiviral immunity as it was shown to be crucial for maintaining KSHV latency. Upon lytic cycle induction with 12-O-tetradecanoyl-phorbol-13-acetate (TPA) in the latently KSHV-infected B-cell lymphoma BC-3 and BCBL-1 cell lines, IFI16 is targeted for proteasomal degradation [20]. This is mediated in part by KSHV late lytic transcripts, and degradation of IFI16 results in increased levels of KSHV lytic proteins and the overall viral genome abundance [20]. Furthermore, Ansari et al. showed by fluorescence in situ hybridization that IFI16 colocalizes with the EBV genome in lymphoblastoid (latency III) and Raji (latency I) cell nuclei [16]. However, despite the extensive studies performed on lytic replicating DNA viruses, recent studies on latent KSHV infections and the observation that IFI16 recognizes the EBV genome, studies on the role of IFI16 in EBV biology are lacking.

The finding that IFI16 is required for maintenance of KSHV latency [20] suggests that IFI16 may also be important for EBV latency. Here, we show for the first time that IFI16 is indeed essential for maintaining latency in the EBV positive, latently I-infected Akata and MUTU-1 cell lines. Knockdown of IFI16 by siRNA-mediated transfection in Akata cells resulted in the efficient reduction of IFI16 mRNA and protein levels, increased EBV lytic gene expression, and increased global EBV viral load. Lytic cycle induction by transforming growth factor β (TGF-β; MUTU-1) and the chemical inducer TPA (Akata) effectively reduced levels of IFI16 protein up to 80% as compared to their uninduced counterparts. This reduction in IFI16 protein correlated to elevated levels of lytic EBV transcripts, increased genome copy number, and a redistribution of IFI16 from the nucleus to the cytoplasm as well. Collectively, these studies demonstrate a vital role for IFI16 during the latent to lytic switch, and provide insight into how EBV interacts with its host.

## Methods

### Cells

The EBV-positive, latently I-infected cell lines Akata and MUTU-1 and the EBV-negative cell line BJAB (all BL cell lines) were cultured in RPMI 1640 GlutaMAX (ThermoFisher, Waltham, MA), supplemented with 10% (vol/vol) fetal bovine serum (FBS; Atlanta Biologicals, GA) and 1% penicillin-streptomycin (ThermoFisher, Waltham, MA). All cell types used for the present studies had routine mycoplasma testing done by a Lonza Myco-Alert kit (LT37–618; Lonza, Walkersville, MD), as per manufacturer’s instructions, and only mycoplasma negative cells were used in these studies.

### Antibodies and reagents

The following antibodies were used in Western blotting and immunofluorescence studies: anti-IFI16 (mouse) and anti-BZLF1; Zebra (mouse) (Santa Cruz Biotechnology, Inc., Santa Cruz, CA); EBV Ea-D p52/p50 and anti-IFI16 (rabbit) (Millipore, Billerica, MA); mouse monoclonal D.1.17.G38 (against gp350/220) was generated in the laboratory [21]. Secondary anti-rabbit-IgG and anti-mouse-IgG antibodies linked to horseradish peroxidase, Alexa Fluor-488, and Alexa Fluor-594 were from KPL, Inc., Gaithersburg, MD, or Molecular Probes, Eugene, OR. 12-O-tetradecanoyl-phorbol-13-acetate (TPA) and phosphonoacetic acid (PAA) were purchased from Millipore-Sigma (Billerica, MA). TGF-β was purchased from Sigma-Aldrich (St. Louis, MO).

### Induction of the EBV lytic cycle

EBV lytic cycle was induced as described in Table 1. The EBV genome abundance was quantitated by real-time DNA PCR with primers specific to EBV nuclear antigen-1 (EBNA1) and samples normalized relative to levels of β-tubulin.

**Table 1:** Details of the inducing agents used in this study

Inducing Agent	Working Concentration	Time in media	Cell Lines
TPA	30 ng/mL	Up to 96 h	Akata & BJAB
TGF-β	5 ng/mL	Up to 96 h	MUTU-1 & BJAB

### siRNA-mediated knockdown and overexpression of IFI16 in B cells

The commercially available SMARTpool siRNA against IFI16 was purchased from Dharmacon (M-020004-01-0010). Akata cells were transfected by electroporation with either siIFI16 or a scrambled siRNA for knockdown studies or with IFI16-FLAG (Addgene; 35,064) or an empty pcDNA3-FLAG vector for overexpression studies, using the Neon transfection system (Life Technologies), according to the manufacturer’s instructions and as described previously with minor modifications (26). Briefly, cells were harvested, washed, and resuspended in resuspension buffer R (provided) at a density of 1.0 × 10^6^ cells/mL. 100 μL of the cell suspension was mixed with 100 pmol siRNA or 3 μg of IFI16 DNA and electroporated using 3 pulses of 1500 V for 10 ms. Following electroporation, cells were replaced into complete medium and incubated at 37 °C with 5% CO_2_ atmosphere. For overexpression studies, 30 ng/mL of TPA was added 4 h after transfection to minimize stress on the cells and incubated for an additional 44 h. For siRNA studies, IFI16 knockdown was confirmed by Western blot and qRT-PCR 48 h post transfection.

### Western blot (WB) analysis

Total cell lysates were prepared by lysing in radioimmunoprecipitation assay (RIPA) buffer (50 mM Tris [pH 8.0], 150 mM sodium chloride, 1.0% NP-40, 0.5% sodium deoxycholate, 0.1% SDS) supplemented with a protease inhibitor cocktail (Sigma) for 30 min on ice. Lysates were then sonicated on ice and centrifuged at 13,000 x g for 10 min at 4 °C, followed by protein estimation using a BCA protein assay reagent kit (Pierce, Rockford, IL). Equal concentrations of protein were separated on 7–12% SDS-PAGE gels, transferred to a nitrocellulose membrane, and probed with primary antibodies as indicated. For detection, species-specific horseradish peroxidase or fluorescence-conjugated (Alexa fluor 488-green or 594-red; Thermo Fisher) secondary antibodies were incubated on membranes for 1 h at room temperature and developed using the SuperSignal West Pico chemiluminescent substrate (Thermo Fisher) or scanned using an AlphaImager system (Alpha Innotech Corporation, San Leonardo, CA).

### Immunofluorescence microscopy (IFA)

Akata, BJAB, and MUTU-1 cells were fixed and permeabilized with ice-cold acetone, washed, and blocked with Image-iT FX signal enhancer (Invitrogen) for 20 min at room temperature. Cells were then incubated with the indicated primary antibody for 1 h at 37 °C, washed, and incubated with either Alexa Fluor-488 or Alexa Fluor-594 (Thermo Fisher) for 30 min at 37 °C. Next, slides were washed and incubated with SlowFade Gold antifade reagent with DAPI (Invitrogen). A Nikon Eclipse 80i fluorescence microscope was used for imaging and analysis was done with Metamorph imaging software.

### RNA purification, reverse transcription, and real-time PCR

RNeasy minikit (Qiagen, Valencia, CA) was used for total cellular RNA isolation as per manufacturer’s instructions. On-column DNase digestion was performed by RNase-free DNase set (Qiagen). Following RNA estimation using a NanoDrop spectrophotometer (Thermo Scientific), 1 μg of RNA was used for reverse transcription with the High-Capacity cDNA reverse transcription kit (Thermo Fisher) according to the manufacturer’s instructions. Gene expression was examined by real-time quantitative reverse transcription PCR (qRT-PCR) using SYBR green chemistry (Applied Biosystems) on an ABI Prism 7500 detection system (Applied Biosystems). The final mRNA gene levels were normalized to the level of β-tubulin and calculated as the delta-delta threshold cycle (ΔΔ*C*_*T*_). Primers used in this study are listed in Table 2.

**Table 2:** Gene functions and mRNA primers used in this study. Primers were designed using EBV genes mapped to the B95.8 genome

EBV Gene	Temporal Class	Function	Forward primer (5′-3′)	Reverse primer (5′-3′)
BZLF1	Immediate early	transcriptional transactivator	ACGACGCACACGGAAACC	CTTGGCCCGGCATTTTCT
BALF2	Early	ssDNA binding protein	GTGAGCTACGCACCCGCCAT	CTGACCGGTTGACTTCG
BZLF2	Late	envelope glycoprotein gp42	CATCGCACTTGTTATTGTTC	CAGACCTCTACATTTGGTTTG
BcRF1	Late	vIL-10	GACAAAGGACGAGGTAGATAA	CTCCAGGTAGAATTGGATCATT
EBNA1	Latent	tethers EBV to sister chromatids in host cell	CCGCAGATGACCCAGGAGAA	TGGAAACCAGGGAGGCAAAT
IFI16	n/a	Host protein	CCCAAAGAAGATCATTGCCATAG	GTTTCGGTCAGCATTCACATC
β-Tubulin	n/a	Host protein	TCCAGATTGGCAATGCCTG	GGCCATCGGGCTGGAT

### Statistical analysis

Data are expressed as means ± standard deviations (SD) from the results of at least three independent experiments (*n* ≥ 3), and the statistical significance was calculated by a two-tailed Student’s *t* test. Values were considered significant if the *P* value was <0.05. Immunoblot densitometric quantifications were done using ImageJ software 1.50.

## Results

### IFI16 knockdown in Akata cells results in the activation of EBV lytic cycle gene expression

**Fig. 1:**
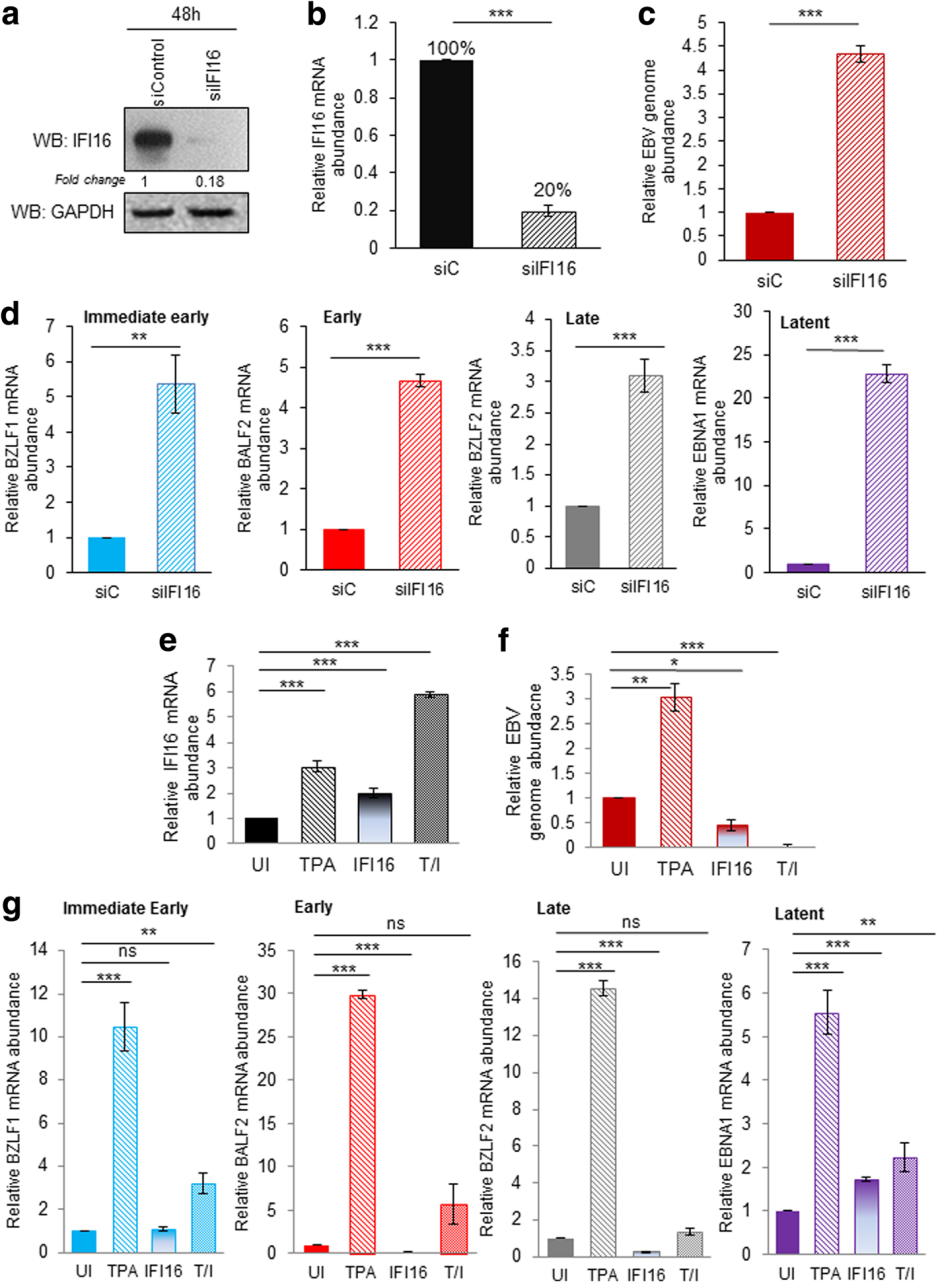
Knockdown of IFI16 in EBV latency I Akata cells results in lytic cycle induction. **a** Immunoblot showing the efficient knockdown of IFI16 in Akata cells at 48 h post siRNA transfection. **b** & **e** Real-time qRT-PCR of IFI16 mRNA levels 48 h after transfection of siRNA (**b**) or IFI16 overexpression (**e**). mRNA levels were normalized against β–tubulin and expressed as relative amounts compared to siC (control) treatments. **c** & **f** Real-time DNA PCR of relative EBV genome abundance. Primers specific to the latent EBNA1 gene were used and the level of DNA was normalized against β-tubulin levels. **d** & **g** Real-time qRT-PCR for gene-specific primers for the four major classes of EBV genes (immediate early, early, late, and latent) 48 h after siRNA transfection (**d**) or IFI16 overexpression and TPA treatment (**g**). mRNA levels were normalized against β-tubulin mRNA levels and data are expressed as the relative amount as compared to the siC treatments. Results are represented as means ± SD of data from three independent experiments and statistical analysis was done with a Student’s *t* test. **, *P* < 0.01; ***, *P* < 0.001

To determine the effect of IFI16 on EBV biology, we down-regulated the gene expression of IFI16 in the latently infected Akata B cells using siRNA (siIFI16). siIFI16 or its control (siC; siControl) were electroporated, incubated up to 48 h, then analyzed for protein, DNA and RNA. By 48 h post transfection, there was approximately an 82% reduction in protein levels and an 80% decrease in IFI16 mRNA (Fig. 1a & b). Interestingly, at the same time that we observed a reduction in IFI16 protein and mRNA levels, there was an increase in total EBV DNA levels as seen by real-time DNA PCR (Fig. 1c), suggesting that loss of IFI16 results in activation of EBV’s lytic cycle.

During EBV lytic cycle induction, the genes expressed occur in a temporal manner. By 48 h post transfection with siIFI16, all four temporal classes, immediate early (IE), early (E), late (L) and latent (Lt) were induced. Specifically, the IE gene and transcriptional transactivator, BZLF1, increased approximately 5.5-fold more than its siControl counterpart (Fig. 1d, blue bars). The E gene BALF2, which is a ssDNA-binding protein and L gene BZLF2, which encodes for an envelope glycoprotein, were also increased 4.5 and 3-fold, respectively (Fig. 1d, red and grey bars). IFI16 knockdown also led to the induction of Lt gene EBV nuclear antigen-1 (EBNA1; Fig. 1d, purple bars), a gene vital for tethering EBV DNA to the sister chromatids of the host cells during cell division and the only viral transcript present in all forms of latencies. Overall, these results conclude that siRNA-mediated knockdown of IFI16 induces lytic reactivation of the latent EBV genome as measured by increased levels of lytic gene expression and total EBV genome abundance.

### Overexpression of IFI16 restricts lytic cycle induction

To further validate IFI16’s involvement in the maintenance of EBV latency, we overexpressed IFI16 in Akata cells followed by lytic cycle induction using the phorbol ester TPA. TPA activates EBV lytic cycle via the NF-κB and AP-1 family of transcription factors, which are activated in response to the protein kinase C and mitogen-activated protein kinase pathways [22–24]. Compared to Akata cells treated with TPA alone and expressing endogenous IFI16, IFI16 overexpressed treatments had approximately 3-fold less EBV abundance as measured by real time DNA PCR (Fig. 1e). Moreover, Akata cells overexpressing IFI16 and incubated with TPA resulted in even less EBV replication than cells transfected with IFI16 alone. This could be a direct result of the excessive IFI16 accumulating on the viral genome and preventing transcription initiation as IFI16 has been reported to do in HSV-1 [19]. When we looked at EBV temporal gene expression, 5.5–30-fold increases were observed in Akata cells induced with TPA (Fig. 1g; striped bars). Contrarily, this effect was reversed when IFI16 was overexpressed and induced with TPA (Fig. 1g; spotted bars), resulting in less than 5-fold induction for all temporal class genes, and suggesting that IFI16 does indeed negatively regulate EBV lytic replication and gene expression. Interestingly, the total amount of IFI16 mRNA present negatively correlated to the total amount of EBV genome and gene expression, whereby the highest levels of IFI16 mRNA had the lowest amount of EBV genome and viral transcripts (Fig. 1f vs. Fig. 1e and g).

### TPA-mediated activation of EBV lytic cycle from latency results in reduced levels of IFI16 protein

**Fig. 2:**
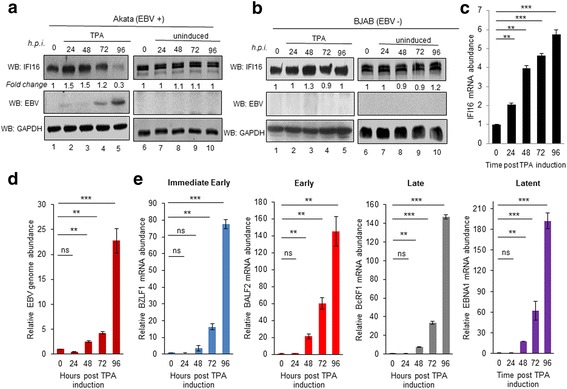
Activation of EBV lytic cycle by TPA in latency I infected Akata cells. **a** Immunoblot of IFI16, EBV (Ea-D p52/50), and GAPDH in the EBV-positive Akata cells either untreated or treated with 30 ng/ml TPA for the indicated time. All bands were normalized to their respective level of GAPDH and the fold change was calculated relative to the uninduced sample. *h.p.i.,* hours post induction. **b** EBV-negative BJAB cells were left uninduced or induced with 30 ng/ml TPA for the indicated time, followed by immunoblotting as done in A. **c** Real time DNA PCR for the relative EBV genome copy number. Primers specific to the latent EBNA1 gene were used and the level of DNA was normalized against β-tubulin. **d** & **e** Real time qRT-PCR of mRNA levels from uninduced to 96 h post TPA induction in Akata cells for IFI16 (**d**) or the four major gene classes of EBV genes in Akata cells (**e**). Total RNA was extracted and mRNA levels analyzed by real-time RT-PCR using gene-specific primers that were normalized against β-tubulin mRNA levels. All values are represented as the relative amount compared to the uninduced control (time zero). Results are presented as ± SD of data from at least three independent experiments and statistical analysis was done with a Student’s *t* test. **, *P* < 0.01; ***, *P* < 0.001; ns, not significant

Our observations that IFI16 KD leads to activation of the lytic cycle and IFI16 overexpression abolishes this effect, suggests that IFI16 has an inhibitory effect on lytic cycle induction. To investigate this further, we chemically induced the lytic cycle using a 96 h treatment of TPA and evaluated its effect on IFI16 expression. As seen in Fig. 2a, treatment with TPA resulted in approximately 70% reduction in IFI16 protein by 96 h post induction (hpi) in Akata cells (lane 5). Ea-D p52/50 (EBV) is an early antigen protein and gene product of BMRF1 and was detected approximately 72 hpi with TPA, verifying activation of the lytic cycle (Fig. 2a, lane 4). To confirm lytic cycle activation, real-time DNA PCR was performed, which steadily increased during the 96 h compared to the uninduced control (Fig. 2c). Despite the significant decrease in IFI16 protein levels by 96 h post induction, a steady increase in IFI16 mRNA occurred (Fig. 2d), perhaps as a result of IFI16 activation in response to reactive oxygen species generated by EBV productive infection [25]. As expected, no lytic cycle induction or decrease in IFI16 was detected in the EBV negative cell line BJAB (Fig. 2b, lanes 1–10) regardless of TPA exposure, or in Akata cells in the absence of TPA (Fig. 2a, lanes 6–10).

In addition to DNA replication, we also analyzed the expression of the different EBV genes by real time qRT-PCR (Fig. 2d). The IE gene product of BZLF1 (ZEBRA) has been reported to be detected as early as 4 hpi by WB and was detected in this study by qRT-PCR at 24 hpi (Fig. 2d, blue bars) [26]. The gene that encodes the ssDNA binding protein, BALF2, was induced by 48 hpi, with the late gene BcRF1 upregulated by 48 hpi and peaking at 96 h (Fig. 2d, red and grey bars). Moreover, a similar trend was observed in the Lt gene EBNA1 (Fig. 2d, purple bars). Along with activation of the lytic cycle, latency III gene expression has been reported during late EBV replication in both B lymphoblasts and epithelial cells, corroborating the results observed here [27].

**Fig. 3:**
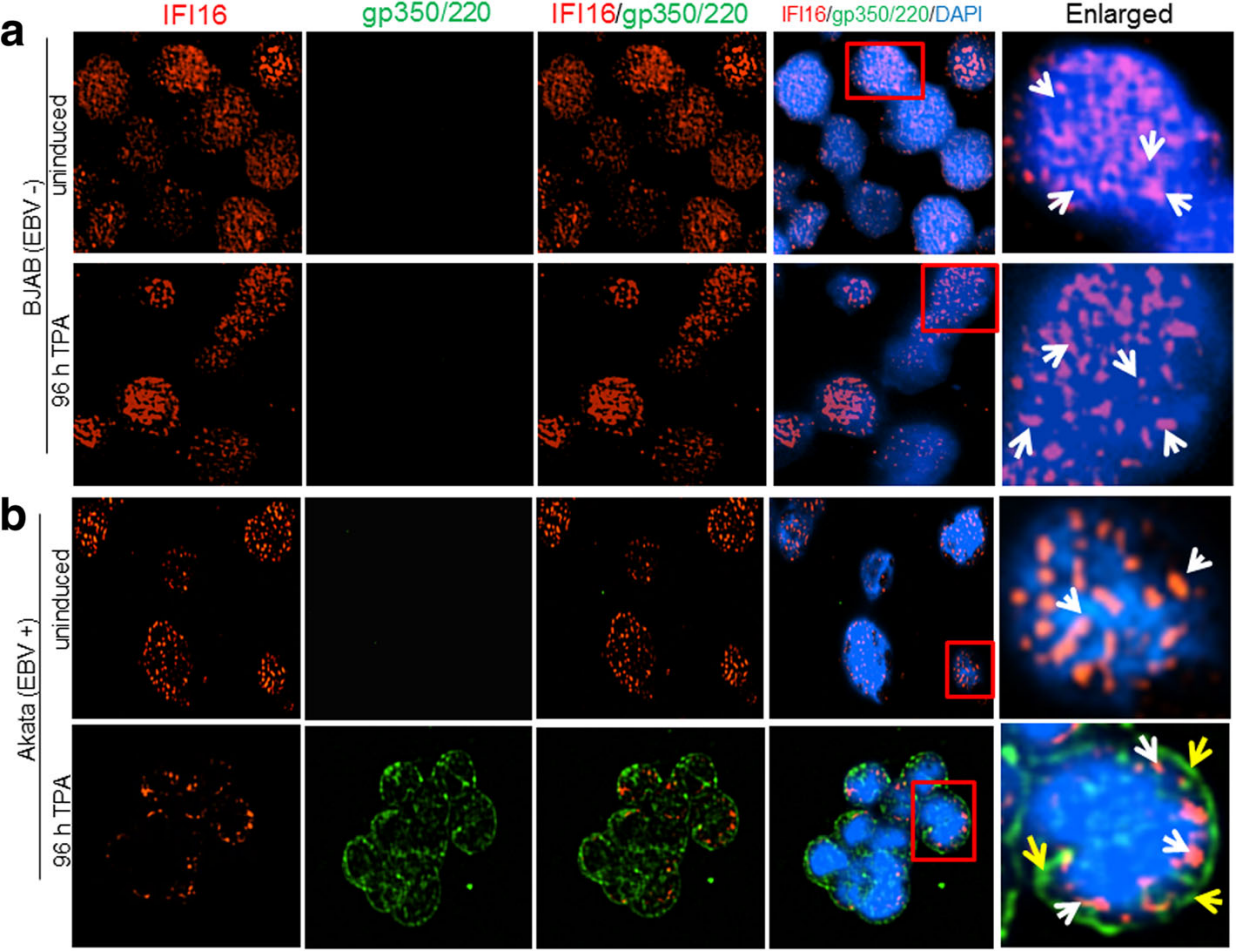
Immunofluorescence analysis of the effect of lytic cycle induction by TPA on IFI16. BJAB (**a**) or Akata (**b**) cells were treated with 30 ng/mL of TPA or left untreated (uninduced) for 96 h. Cells were harvested, plated onto 10-well slides, fixed with ice-cold acetone, thoroughly washed, and blocked for 30 min at room temperature. Slides were stained with primary antibodies against the EBV glycoprotein gp350/220 (green) to visualize infected cells undergoing lytic replication and anti-IFI16 (red). Cell nuclei were visualized by DAPI staining (blue). Magnification, 40X. Red boxes are enlarged in the far right panels with white arrows indicating IFI16 puncta and yellow arrows showing gp350/220 puncta. A representative experiment out of three is shown

Previous studies have reported that IFI16 translocates to the cytoplasm in all EBV-positive, latently infected cells, as a result of genome recognition by IFI16 [28]. To determine if there was also a redistribution and/or change in the abundance of IFI16 in Akata cells following lytic cycle activation, we performed immunofluorescence analysis (IFA). Akata or BJAB cells were treated with TPA for 96 h and stained for IFI16 and the EBV envelope glycoprotein gp350/220, which was used as a marker of lytic activity. As seen in Fig. 3b, induced Akata cells express EBV ​gp350/220 in both cytoplasm and nucleus, although it is predominantly perinuclear. Interestingly, in the same cells express​ing EBV gp350/220, ​a reduction in IFI16 ​ levels was observed, which corroborated the Western blot results shown in Fig.2. Similar to the IFI16 observed in the uninduced Akata cells (Fig. 3b, top panel) BJAB had IFI16 expressed abundantly throughout the nucleus, independent of exposure to TPA (Fig. 3a, top and bottom panels). These results confirm that cells undergoing lytic EBV re​activation display a reduced IFI16 level.

### Induction of latency I infected MUTU-1 cells using TGF-β results in decreased IFI16 protein

**Fig. 4:**
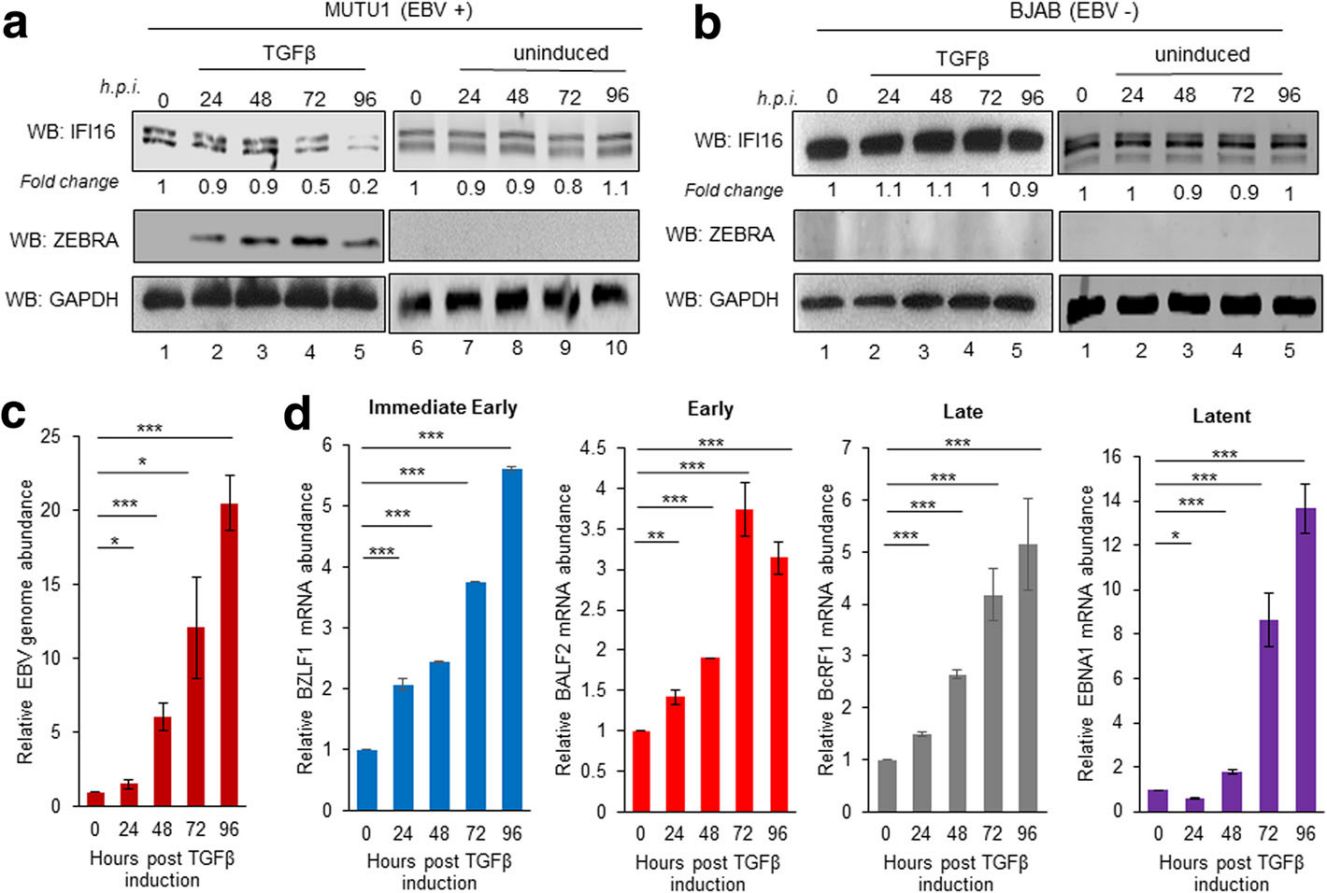
EBV lytic cycle induction by TGF-β in latency I infected MUTU-1 cells. MUTU-1 cells were either uninduced (time zero) or treated with TGF-β (5 ng/mL) for the indicated time. **a** Immunoblots for IFI16 (top), ZEBRA (BZLF1; middle), or GAPDH (bottom) following induction. All bands were normalized to GAPDH with the fold change calculated relative to the uninduced sample. **b** EBV-negative BJAB cells were treated with TGF-β similar to MUTU-1 cells or left untreated and immunoblotted for IFI16, ZEBRA, and GAPDH. **c** Real time DNA PCR for the relative EBV genome abundance. Primers specific to the latent EBNA1 gene were used and the level of DNA was normalized against the β-tubulin level. **d** Real time qRT-PCR for mRNA levels in uninduced to 96 h post TGF-β induction in MUTU-1 cells. Total RNA was extracted and mRNA levels analyzed by real-time qRT-PCR using gene-specific primers to EBV temporal class genes. mRNA values were normalized against β-tubulin mRNA levels. All values are represented as the relative amount compared to the uninduced (time zero) control. Results are presented as ± SD of data from three independent experiments and statistical analysis was done with a Student’s *t* test. *, *P* < 0.05; **, *P* < 0.01; ***, *P* < 0.001; ns, not significant

Depending on the cells studied, EBV lytic cycle can be intentionally induced by various stimuli, including anti-immunoglobulin cross-linking, chemical agents like sodium butyrate and TPA, Ca^2+^ ionophores, or TGF-β. It is also known that some chemical inducers like sodium butyrate and TPA can induce cell death [29]. Therefore, we utilized another BL cell line, MUTU-1 (Lat. I) induced with TGF-β to not only confirm the results observed in Akata cells, but also to minimize any stress-induced death that may be occurring to the cells. For these studies, MUTU-1 cells were incubated with TGF-β for up to 96 h to observe the effect of lytic induction on IFI16 expression. As seen in Fig. 4a, there was a 50% decrease in IFI16 protein by 72 hpi, which continued to decrease up to 80% by 96 hpi. Lytic cycle activation was confirmed by both the presence of ZEBRA (protein product of BZLF1), as well as the total abundance of EBV genome DNA as measured by real time DNA PCR (Fig. 4a middle panel & 4C).

To assess the effect of TGF-β on EBV lytic gene expression, real-time RT-PCR was performed using RNA collected from MUTU-1 cells either untreated or at different time points following TGF-β stimulation. The IE BZLF1 steadily increased during the 96 h, peaking at 5.5-fold by 96 h, and E gene BALF2 also increased following TGF-β, peaking at 72 hpi before beginning to decrease at 96 h (Fig. 4d, blue and red bars). Similar to BZLF1, late and latent genes BcRF1 and EBNA1 peaked at 96 hpi at 5 and 14-fold increases, respectively (Fig. 4d, grey and purple bars).

**Fig. 5:**
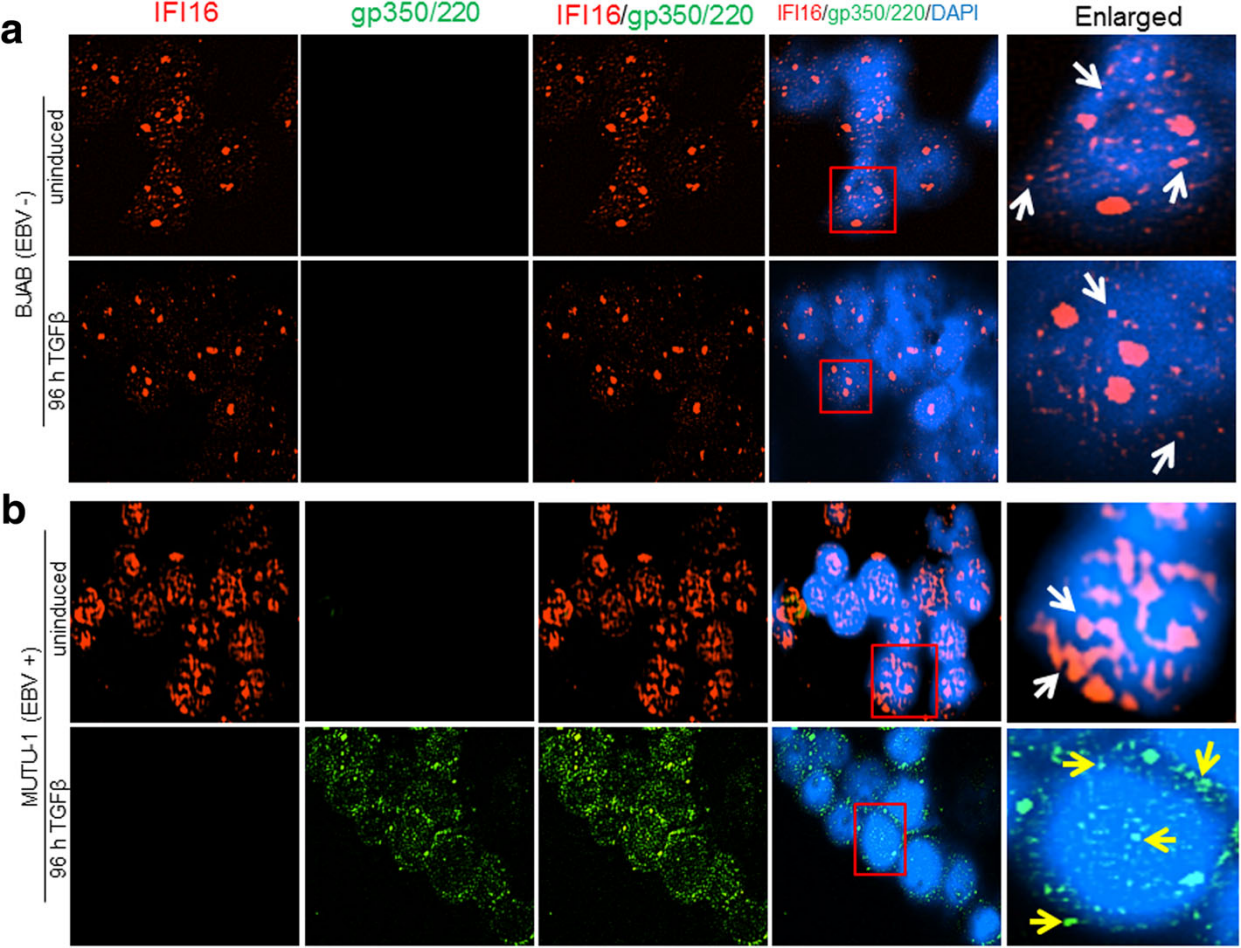
TGF-β-induced lytic cycle in latency I infected MUTU-1 cells results in decreased levels of IFI16 protein. BJAB (**a**) or MUTU-1 cells (**b**) were induced with TGF-β (5 ng/mL) or left untreated for 96 h. 96 h after induction, cells were harvested and prepared for IFA as described previously (Fig. 3). Slides were stained with primary antibodies to IFI16 (red) and EBV glycoprotein gp350/220 (green). Cell nuclei were visualized by DAPI staining (blue). Magnification, 40X. Red boxes are enlarged in the far right panels with white arrows indicating IFI16 puncta and yellow arrows showing gp350/220. A representative experiment out of three is shown

To assess if lytic EBV reactivation results in changes of IFI16 in MUTU-1 cells, we also performed IFA. 96 hpi with TGF-β, MUTU-1 and BJAB cells were harvested and IFI16 analyzed in cells expressing gp350/220. As seen in Fig. 5b, MUTU-1 cells treated with TGF-β stained intensely for gp350/220, confirming lytic cycle activation. In the same cells that stained for gp350/220, we did not observe IFI16 (Fig. 5b), which corroborates with the 80% reduction in IFI16 protein seen by WB following TGF-β treatment (Fig. 4a, lane 5). No reduction or relocalization in IFI16 was observed in our uninduced MUTU-1 cells or in BJAB cells independent of TGF-β exposure (Fig. 5b top panel; Fig. 5a, top and bottom panels). These results provide additional evidence that the changes observed in IFI16 levels are specific to cells undergoing EBV lytic activation.

**Fig. 6:**
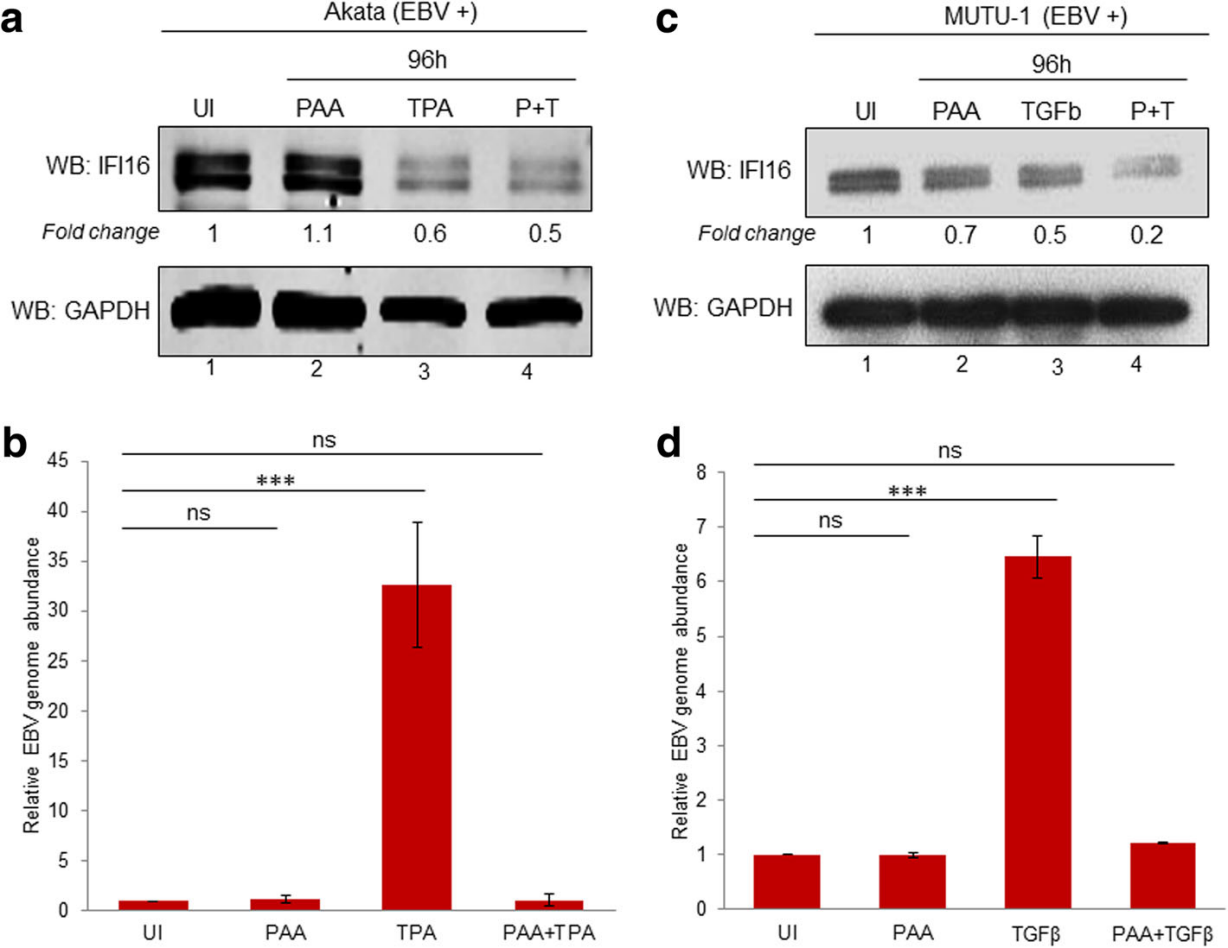
IFI16 protein reduction is not dependent on late lytic transcripts. **a** Akata cells were left untreated (lane 1), incubated with 100 μg/mL PAA for 96 h (lane 2), with 30 ng/mL TPA for 96 h (lane 3), or with 100 μg/mL PAA for 1 h followed by the addition of 30 ng/mL TPA for an additional 96 h (lane 4), then immunoblotted for anti-IFI16 or anti-GAPDH. **b** MUTU-1 cells were treated the same as in A, except with 300 μg/mL of PAA and with 5 ng/mL of TGF-β in place of TPA. **b** & **d** Real time DNA PCR was performed to analyze relative EBV genome abundance in Akata (**b**) or MUTU-1 (**d**) cell lines. Primers specific to the latent EBNA1 gene were used and the level of DNA was normalized against the β-tubulin level. Results are presented as ± SD of data from three independent experiments and statistical analysis was done with a Student’s *t* test. ***, *P* < 0.001; ns, not significant

In order to gain insight into the mechanism of how IFI16 protein is decreasing, we treated both Akata and MUTU-1 cell lines with the EBV late lytic transcript inhibitor PAA. PAA is a non-toxic, viral DNA polymerase inhibitor that specifically prevents successful EBV genome replication, without affecting IE or E gene synthesis [30]. As seen by WB in fig. 6a, IFI16 levels were reduced by 40% in the presence of TPA alone and up to 50% with PAA and TPA together in Akata cells. Relative EBV genome abundance was analyzed by real time DNA PCR to validate PAA effectiveness and ensure EBV replication in the TPA-treated sample (Fig. 6b). At the same time, MUTU-1 cells were treated with TGF-β alone or TGF-β and PAA (Fig. 6c & d). Comparable to Akata cells, PAA treatment of MUTU-1 was unable to restore IFI16 protein levels to levels of its uninduced counterpart (Fig. 6c), which was not a result of EBV replication given that no increase in EBV genome abundance was observed in the PAA alone treatments (Fig. 6c & d). Furthermore, MUTU-1 cells in the presence of PAA alone resulted in an additional decrease in IFI16 protein levels, especially compared to Akata cells treated with PAA alone (Fig. 6c, lanes 2 vs. 1 and lanes 4 vs. 3). This could be due to subtle differences between Akata and MUTU-1 cell lines or PAA having a non-specific inhibition on IE and E genes, and possibly even cellular polymerases in MUTU-1 cells as a result of the higher concentration of PAA used in these experiments compared to the concentration used in the Akata experiments (300 μg/mL vs. 100 μg/mL). Additionally, Inman et. al reported that EBV positive cells undergoing lytic replication are protected from apoptosis-mediated cell death and treatment with PAA blocked this protection, suggesting that the decrease in IFI16 observed in these studies is a result of lytic cells undergoing apoptosis in the presence of PAA [31]. Overall, treatment with PAA did not result in increased levels of IFI16 protein. This suggests that the decrease is due to EBV IE or E genes products (proteins, miRNA, or lincRNA) either directly or indirectly; however, which EBV gene product(s) mediating IFI16 requires further study and is beyond the scope of the current manuscript.

## Discussion

Antiviral immunity against foreign, invading pathogens is vital for ensuring the survival of the infected host. Some viruses such as herpesviruses, however, are never cleared, but instead alternate between a lytic and latent state throughout the life of the infected individual. During lytic infection, progeny virus are produced and are recognized by the immune system, resulting in a myriad of cellular responses against the virus.

To overcome host detection, EBV has evolved a complex set of latencies unique to EBV. It’s during these latencies that EBV-associated malignancies such as BL, nasopharyngeal carcinoma, and lymphoproliferative diseases (amongst others) arise. Unfortunately, the only available treatments for such diseases include chemotherapy and immunotherapy, which, if effective, can have significant toxic side effects and leave the patients permanently deformed [32–34]. Therefore, identifying host proteins that are involved in EBV infection is crucial for developing new strategies to combat viral-induced diseases.

Besides its role as an integral part of the inflammasome during dsDNA infection, IFI16 has been reported to have diverse gene regulatory and chromatin remodeling functions during HSV-1, HCMV, HPV, and KSHV infections [17, 19, 20, 35]. IFI16 acts as a negative regulator in its ability to bind to the promoters of all temporal gene classes in HSV-1 while inhibiting transcription factor binding such as Oct1, TBP, and RNA Pol II [19]. Moreover, IFI16 restricts viral DNA replication in HPV in part by its ability to epigenetically modify viral promoters [17]. Gariano et al. showed that over-expression of IFI16 in human embryo lung fibroblasts resulted in decreased viral yield and total DNA abundance and that this was due to IFI16’s ability to bind to the HCMV promoter UL54, ultimately blocking Sp-1-like binding sites and preventing both early and late viral gene expression [18]. Additionally, silencing of IFI16 using siRNA reversed these effects, enhancing viral replication, which are similar to the results observed in our EBV experiments [18]. In the present study, siRNA-mediated KD of IFI16 in Akata cells results in lytic reactivation of EBV, as measured by increases in the viral genome and all temporal class gene expression (Fig. 1c & d). Contrarily, overexpression of IFI16 reversed these effects, leading decreased EBV replication and gene expression compared to samples treated with TPA alone (Fig. 1f & g). These results suggest that IFI16 is involved in lytic cycle suppression, and therefore, may directly contribute to disease progression as all EBV-associated malignancies occur during latency.

The activation of lytic genes was observed in both Akata cells induced with TPA and MUTU-1 cells induced with TGF-β, with Akata cells displaying statistically higher levels of induction (Fig. 2c and d vs. Fig. 4c and d). Differences in viral genome abundance, lytic gene expression, and IFI16 localization observed in our IFA studies (Figs. 3 and 5) could be due to i) the origin of the cell lines (a Japanese BL vs. a Kenyan BL), ii) the effectiveness of the inducer (TPA vs. TGF-β), iii) the ability of the cells to respond to their respective inducers over time (possible loss of sensitivity of cells to respond after extensive passages), or iv) the spontaneous loss of virus from the cells [36–38].

To date, IFI16 degradation has only been reported to occur in HSV-1 de novo infection [39–41] and lytic reactivation of KSHV-infected cells [20]. Recently, it was reported that IFI16 is polyubiquitinated and degraded via the proteasome-mediated pathway in the KSHV latently infected B lymphoma cell lines BCBL-1 and BC3 [20]. This degradation was shown to occur at least in part by late lytic transcripts, as treatment with PAA restored IFI16 protein levels to that of uninduced controls [20]. Using similar approaches with our EBV models, we did not observe any significant increase in IFI16 protein despite blocking protein degradation with MG132 (data not included) or the transcription of EBV late lytic transcripts with PAA (Fig. 6a-d). Rather, our results suggest that the changes in IFI16 protein may be due to IE or E genes, or perhaps a combination of the two. Furthermore, the fact that IFI16 mRNA abundance steadily increases from 0 to 96 h post induction with TPA (Fig. 2d) despite the reduction observed in protein levels, suggests that the reduction is occurring via a post-translational event. Nonetheless, it is clear that KSHV and EBV use distinct mechanisms in response to foreign, invading pathogens.

In these studies, we found that IFI16 protein levels are reduced during reactivation of the EBV lytic cycle. EBV is associated with >90% of endemic BL cases, lymphoproliferative diseases, and is implicated in gastric carcinoma, the fifth leading cause of cancer morbidity in the world [42]. IFI16 has previously been shown to be required for the establishment of KSHV latency and acts as a negative regulator of HSV-1 viral gene expression. Our studies, for the first time, implicate IFI16 as a requirement for the maintenance of EBV latency and suggest that IFI16 gets targeted post-translationally for degradation by a currently unknown mechanism.

## Conclusions

The findings presented here provide evidence that EBV uses IFI16 to maintain its latent state. We show that IFI16 protein levels decrease following lytic cycle reactivation, implying an important role for IFI16 in the maintenance of latency and as a negative regulator of lytic cycle induction. Importantly, identification of IFI16 in the regulation of EBV latency will provide future insight into treating EBV-related malignancies and the cellular-induced immune control mechanisms that control them.

## Abbreviations

ASC: Apoptosis-associated speck-like protein containing CARD
BL: Burkitt’s lymphoma
E: Early
EBNA1: EBV nuclear antigen-1
EBV: Epstein-Barr Virus
HCMV: Human cytomegalovirus
hpi: H post induction
HPV: Human papilloma virus
IE: Immediate early
IFA: Immunofluorescence analysis
IFI16: Interferon-γ-inducible 16
KSHV: Kaposi’s sarcoma associated herpesvirus
L: Late
Lat: Latency
Lt: Latent
PAA: Phosphonoaceitic acid
siC: Si control
siRNA: short interferon RNA
TGF-β: Transforming growth factor-β
TPA: 12-O-tetradecanoyl-phorbol-13-acetate
